# Melatonin supplementation in the subacute phase after ischemia alleviates postischemic sleep disturbances in rats

**DOI:** 10.1002/brb3.2366

**Published:** 2021-09-14

**Authors:** Shu‐Mei Hao, Zhi‐Gang Zhong, Wei‐Min Qu, Zhi‐Li Huang, Feng‐Yan Sun, Mei‐Hong Qiu

**Affiliations:** ^1^ Department of Neurobiology Institute for Basic Research on Aging and Medicine School of Basic Medical Science Fudan University Shanghai China; ^2^ Department of Pharmacology School of Basic Medical Science State Key Laboratory of Medical Neurobiology and MOE Frontiers Center for Brain Science Fudan University Shanghai China

**Keywords:** circadian rhythm, ischemic stroke, melatonin, sleep disorder, sleep‐wake

## Abstract

**Background:**

Sleep disorders are highly prevalent among stroke survivors and impede stroke recovery. It is well established that melatonin has neuroprotective effects in animal models of ischemic stroke. However, as a modulator of endogenous physiological circadian rhythms, the effects of melatonin on poststroke sleep disorders remain unclear. In the present study, we investigated how melatonin delivered intraperitoneally once daily in the subacute phase after stroke onset, influencing neuronal survival, motor recovery, and sleep–wake profiles in rats.

**Methods:**

Transient ischemic stroke in male Sprague–Dawley rats was induced with 30 min occlusion of the middle cerebral artery. Melatonin or vehicle was delivered intraperitoneally once daily in the subacute phase, from 2 to 7 days after stroke. Electroencephalogram and electromyogram recordings were obtained simultaneously.

**Results:**

Compared to the effects observed in the vehicle‐treated ischemic group, after 6 daily consecutive treatment of melatonin at 10 mg/kg starting at ischemic/reperfusion day 2, the infarct volume was significantly decreased (from 39.6 to 26.2%), and the degeneration of axons in the ipsilateral striatum and the contralateral corpus callosum were significantly alleviated. Sensorimotor performances were obviously improved as evidenced by significant increases in the latency to falling off the wire and in the use of the impaired forelimb. In addition to those predictable results of reducing brain tissue damage and mitigating behavioral deficits, repeated melatonin treatment during the subacute phase of stroke also alleviated sleep fragmentation through reducing sleep‐wake stage transitions and stage bouts, together with increasing stage durations. Furthermore, daily administration of melatonin at 9 a.m. significantly increased the nonrapid eye movement sleep delta power during both the light and dark periods and decreased the degree of reduction of the circadian index.

**Conclusions:**

Melatonin promptly reversed ischemia‐induced sleep disturbances. The neuroprotective effects of melatonin on ischemic injury may be partially associated with its role in sleep modulation.

## Abstract 2

Although sleep disturbances are highly prevalent among stroke survivors and impede post‐stroke recovery, they are still insufficiently considered in the prevention and management of stroke patients. Little attention has been paid to its physiological consequences of melatonin of regulating circadian rhythms on stroke subjects. Our results showed that in addition to the predictable effects on reducing brain damage and mitigating behavioral deficits, repeated melatonin treatment during the sub‐acute phase of stroke also ameliorated circadian rhythm disruption, alleviated sleep fragmentation, and increased sleep depth in ischemic rats. The findings revealed the ability of melatonin to quickly and promptly reverse ischemia‐induced sleep disturbances; a new perspective on how melatonin protect the brain against ischemia injury; and a suitable strategy for improving clinical outcomes in acute stroke victims.

## 1 INTRODUCTION

Ischemic stroke is a major disabling disease, which endangers human health and seriously affects the quality of life of affected patients (Dabrowska‐Bender et al., 2017; Khaku & Tadi, 2020; Kim et al., 2017). The incidence of sleep disorders in stroke patients is higher than that in the general population (Chen et al., 2015; Glozier et al., 2017; Kim et al., 2017; Seiler et al., 2019). There is a more than 50% prevalence of sleep‐disordered breathing (SDB) after stroke(Chen et al., 2015; Hermann & Bassetti, 2009), and approximately 20%−40% of stroke survivors suffer from sleep‐wake disorders, such as insomnia, hypersomnia, circadian rhythm disorders, and sleep‐related movement disorders (Leppavuori et al., 2002; Pajediene et al., 2020). Sleep disturbances are not only a risk factor for stroke but also further exacerbate the deleterious effects of stroke. For example, clinical observations suggest that sleep disorders further aggravate cognitive dysfunction, as well as trigger emotional changes, lethargy, and fatigue, thereby hindering the recovery of stroke patients, affecting stroke outcomes, and increasing recurrence rates (Bassetti et al., 2020; Duss et al., 2017; Hermann & Bassetti, 2009; Sterr et al., 2018). Therefore, active interventions for sleep disturbances after stroke are needed; however, sleep‐wake disturbances are often neglected in stroke patients.

Given the high incidence of sleep disorders in stroke patients and their impact on poststroke recovery, medications that improve sleep may be beneficial for poststroke rehabilitation. Unfortunately, systematic studies supporting this hypothesis are lacking. The widely known harms and adverse effects of benzodiazepines may worsen SDB and even neurological deficits (Feld & Diekelmann, 2015), outweighing their sleep‐promoting benefits. On the basis of data from animal models, nonbenzodiazepine hypnotics such as zolpidem that are relatively free of cognitive and muscle‐relaxant effects, may have beneficial effects in stroke recovery (Bomalaski et al., 2017; Hiu et al., 2016). However, case‐control studies have indicated that zolpidem is dose‐dependently associated with ischemic stroke risk (Huang et al., 2013), indicating that it may cause stroke recurrence, which has limited its use as a remedy for stroke‐induced insomnia.

Previous studies have shown that the levels of melatonin and its metabolite, 6‐sulfatoxymelatonin, in serum and urine are lower in patients with acute ischemic stroke than in healthy controls (Fiorina et al., 1999; Ritzenthaler et al., 2013). As endogenous melatonin is involved in the circadian control of the sleep‐wake cycle and has sleep‐promoting effects (Melo et al., 2017), decreased melatonin levels may explain why sleep disorders are commonly observed in poststroke patients. Therefore, restoring melatonin levels may help to mitigate poststroke sleep problems. Numerous experimental models of brain ischemia have demonstrated that melatonin is highly effective in reducing infarct size, improving neuronal survival and neurogenesis, and reducing mortality (Ramos et al., 2020). Such neuroprotective effects of melatonin are thought to be related to its antioxidant, anti‐apoptosis, and anti‐inflammatory effects (Andrabi et al., 2015; Balduini et al., 2012; Xie et al., 2017; Yang et al., 2015). However, little attention has been paid to its physiological consequences of regulating circadian rhythms on stroke subjects, either in animal models or in clinical trials. Hence, the aim of the present study was to investigate the effects of melatonin supplementation during the sub‐acute phase of stroke on sleep disturbances in rats with transient middle cerebral artery occlusion (MCAO).

## 2 MATERIALS AND METHODS

### 2.1 Animals, experimental procedures, and treatments

**FIGURE 1:**
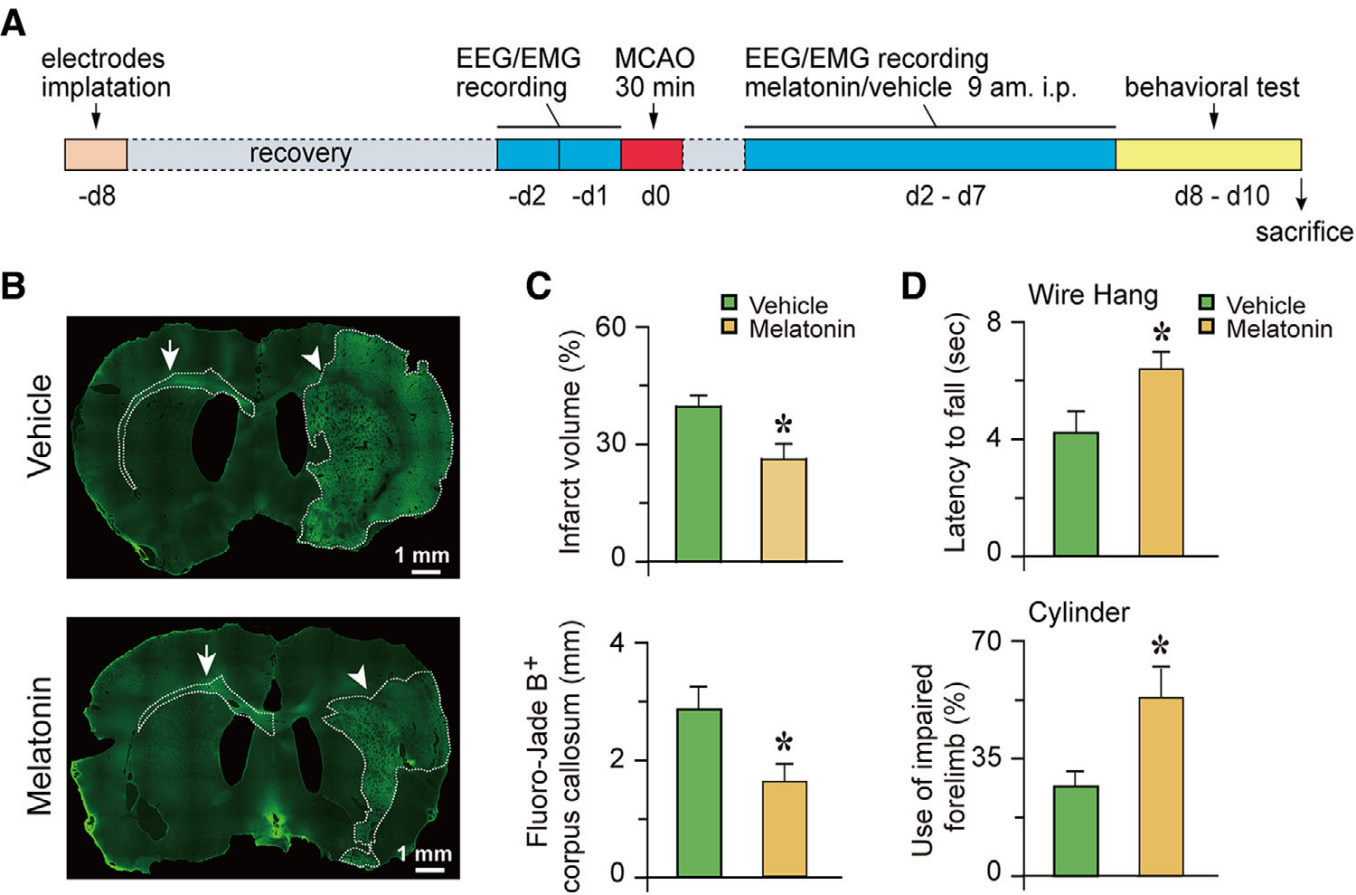
Melatonin reduces infarct volume and improves motor functions after ischemic injury. (a) Timeline of procedures for treatments in rats. (b) Representative Fluoro‐Jade B staining in coronal sections of rats at day 10 after MCAO. The bright green areas indicate infarct areas. (c) Melatonin significantly reduced both the infarct volume and the length of the degenerated contralateral corpus callosum of ischemic injured rats at day 10 after MCAO (*n* = 6). (d) The wire test and cylinder test revealed that the latency to fall and the use of impaired forelimbs, respectively, in melatonin‐treated rats were significantly increased compared to those of the vehicle‐treated group at day 10 after MCAO (*n* = 12). Data are presented as the mean ± SE, *n* = 6−12. *p < 0.05, assessed by two‐tailed unpaired Student's *t*‐test

Male Sprague–Dawley rats, weighing 250−300 g, were obtained from Shanghai Experimental Animal Center (Shanghai, China). Rats were individually housed under a 12‐h/12‐h light/dark cycle (lights on at 7 a.m.), at an ambient temperature of 22°C ± 0.5°C and a relative humidity of 60% ± 2%. All rats were provided free access to food and water ad libitum. All experimental protocols were approved by the Committee on the Ethics of Animal Experiments in the School of Basic Medical Science at Fudan University (Permit number: 20170223‐043). Every effort was made to minimize the number of experimental animals used and any pain and discomfort experienced by the subjects. Baseline electroencephalograms (EEGs) and electromyograms (EMGs) of the rats were recorded before the MCAO procedure, and EEGs/EMGs were continuously recorded for a further 6 days from ischemic/reperfusion day 2 (I/R d2) to I/R d7 after MCAO. During these 6 days, freshly prepared melatonin or vehicle was intraperitoneally administered to the rats at 9 a.m. each day. Neurobehavioral tests were performed on rats during I/R d8–d10 after MCAO. Following completion of the experiment, the rats were euthanized, and their brain tissues were collected and assessed for brain damage (Figure 1a).

### 2.2 Sleep‐wake recordings and analysis

Rats were anesthetized with chloral hydrate (10% in saline, 360 mg/kg, i.p.) and implanted with electrodes for EEG/EMG recordings as described previously (Qiu et al., 2010). EEG/EMG recordings and analysis were performed as described in the Supporting Information. The circadian amplitude of wakefulness was calculated using the following formula: circadian index of wakefulness (CI_wakefulness_) = (mean_dark_ – mean_light_)/mean_24h_ (Qiu et al., 2015).

### 2.3 Transient focal ischemia

Transient focal ischemia was produced by 30 min of MCAO. The procedure is described in the Supporting Information. After recovering from anesthesia, the rats were returned to the recording chamber for EEG/EMG acquisition, and during that period, they were provided food and water ad libitum.

### 2.4 Melatonin treatment

Melatonin (Best Life Inc., Union City, CA, USA) was dissolved in DMSO and diluted with 0.9% saline to a final concentration of 5% DMSO. Melatonin was administered intraperitoneally at a dose of 10 mg/kg at 9 a.m. each day from 2 to 7 days after ischemic stroke. The rats in the vehicle group were injected with 5% DMSO in saline.

### 2.5 Preparation of brain sections

The rats were anesthetized with 10% chloral hydrate and were perfused intracardially with saline followed by 4% paraformaldehyde in 0.1 mol/L of phosphate buffer (pH = 7.4). Brains were removed and postfixed in 4% paraformaldehyde for 4−6 h and were then successively equilibrated in 20 and 30% sucrose solutions. Serial coronal sections were sliced on a freezing microtome (Model 820‐II; Leica, Germany) at a thickness of 30 μm and were stored at −20°C in a cryoprotectant solution.

### 2.6 Fluoro‐Jade B and immunohistochemical staining

Fluoro‐Jade B (4 mg/L in 0.1% acetic acid, Millipore, Billerica, MA, USA) staining and immunohistochemical staining for SMI32 (1:500; ab27375, Abcam, Cambridge, UK) were performed as described in the Supporting Information.

### 2.7 Neurological function tests

The wire hang test, cylinder test, and rotarod test were assessed in a blinded manner as described in the Supporting Information.

### 2.8 Statistical analysis

Animals were randomly assigned into two groups and were numbered individually. Data analysis was performed by investigators who were blinded to the experimental treatments and using GraphPad Prism for Windows (GraphPad Software, San Diego, CA, USA). All data are presented as the mean ± SE. Statistical significance was assessed via the two‐tailed Student's *t*‐test, with *p* < 0.05 taken as the threshold of significance. The inclusion and exclusion criteria are described in the Supporting Information.

## 3 RESULTS

### 3.1 Neuroprotective effects of melatonin in MCAO rats

Neuronal death was revealed by Fluoro‐Jade B, a marker of neurodegeneration. As expected, in the vehicle‐treated group, transient MCAO for 30 min induced extensive infarctions, including within the striatum and cortex on I/R d10. Following six daily consecutive treatments of melatonin at 10 mg/kg starting at I/R d2, the infarct volume was significantly decreased compared to that of the vehicle‐ischemic group (26.2% ± 4.0 % vs. 39.6% ± 2.9%, *p* = 0.022; Figure 1b & c, upper panel). Fluoro‐Jade B staining was also detected in the contralateral corpus callosum. We measured the length of the Fluoro‐Jade‐B‐labeled contralateral corpus callosum and found that it was significantly shortened in the melatonin‐treated ischemic group compared to that in the vehicle‐treated group (1.6 ± 0.3 mm vs. 2.9 ± 0.4 mm, *p* = 0.041; Figure 1c, lower panel).

**FIGURE 2:**
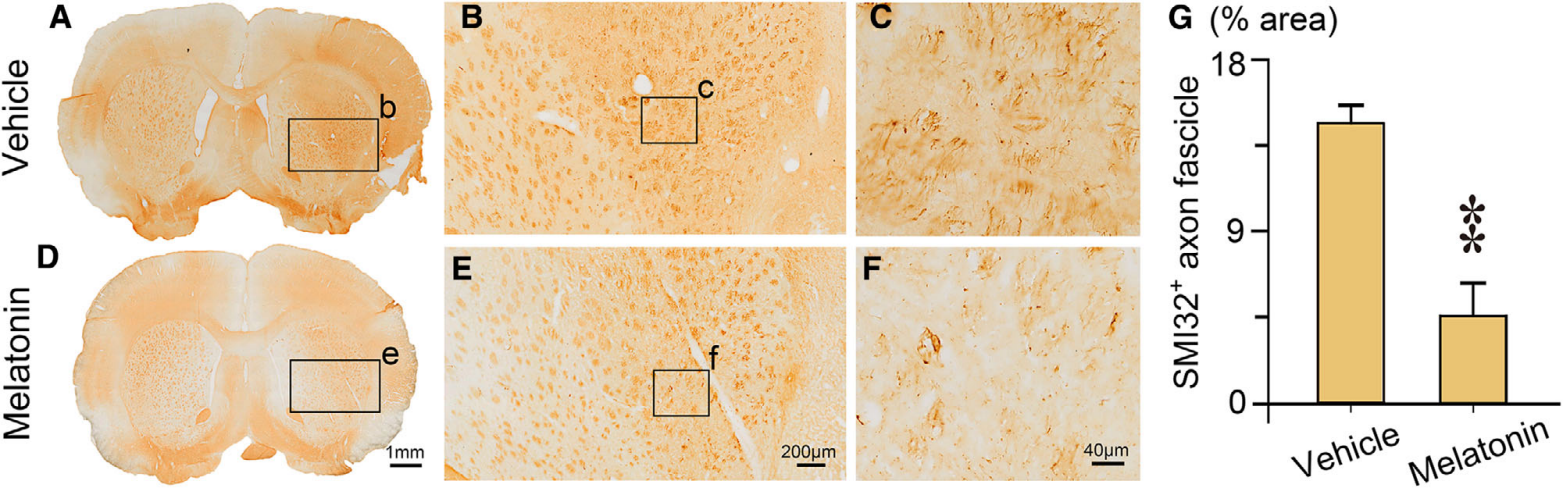
Melatonin decreases demyelination after MCAO. Representative photomicrographs of SMI32 immunostaining in coronal sections of vehicle or melatonin treated rats at day 10 after MCAO. (b and e) Magnified views of the rectangular boxes marked in (a) and (d), respectively. (c and f) Magnified views of the rectangular boxes marked in (b) and (e), respectively. (g) quantification (%) of SMI‐32 immuno‐positive axon fascicles in the infarct striatum of vehicle (*n* = 5) and melatonin (*n* = 6) treated rats at day 10 after transient ischemia. Data are presented as the mean ± SE, **p < 0.01, assessed by two‐tailed unpaired Student's *t*‐test

Sensorimotor outcomes assessed at day 8−10 after MCAO showed no significant difference in the motor coordination of rats between the two treatment groups, as determined by the rotarod test (170.7 ± 23.6 s vs. 158.9 ± 21.9 s, *p* = 0.719; Figure S1). In contrast, there were significant increases in the latency to fall off the wire (6.3 ± 0.6 s vs. 4.2 ± 0.7 s, *p* = 0.030) and in the use of the impaired forelimb (53.1% ± 8.7% vs. 26.7% ± 3.5%, *p* = 0.005) in melatonin‐treated ischemic rats compared to vehicle‐treated ischemic rats (Figure 1d). This finding indicates that neurological deficits induced by cerebral ischemia were significantly less pronounced after melatonin treatment. To further investigate whether melatonin affects neuronal degeneration following ischemic injury, we immunostained the brain tissue with SMI32, a neuronal cytoskeletal marker that recognizes nonphosphorylated neurofilaments, to assay axonal injury after MCAO (Suenaga et al., 2015). Specifically, MCAO led to demyelination of axons, as shown by the intense SMI32 immunoreactivity on axon fascicles in the infarct area of the ipsilateral striatum (Figure 2a–c). In the melatonin‐treated group, SMI32 expression was significantly decreased in the infarct striatum at day 10 after transient ischemia (Figure 2d–f). Quantification (%) of SMI32 immuno‐positive axon fascicles were significantly decreased in the melatonin treated group (4.6% ± 1.8% vs. 14.5% ± 0.9%, p = 0.0003; Figure 2g), indicating that melatonin preserved myelin integrity, facilitating the recovery of white matter after ischemia.

### 3.2 Effects of melatonin on vigilance states of MCAO rats

**FIGURE 3:**
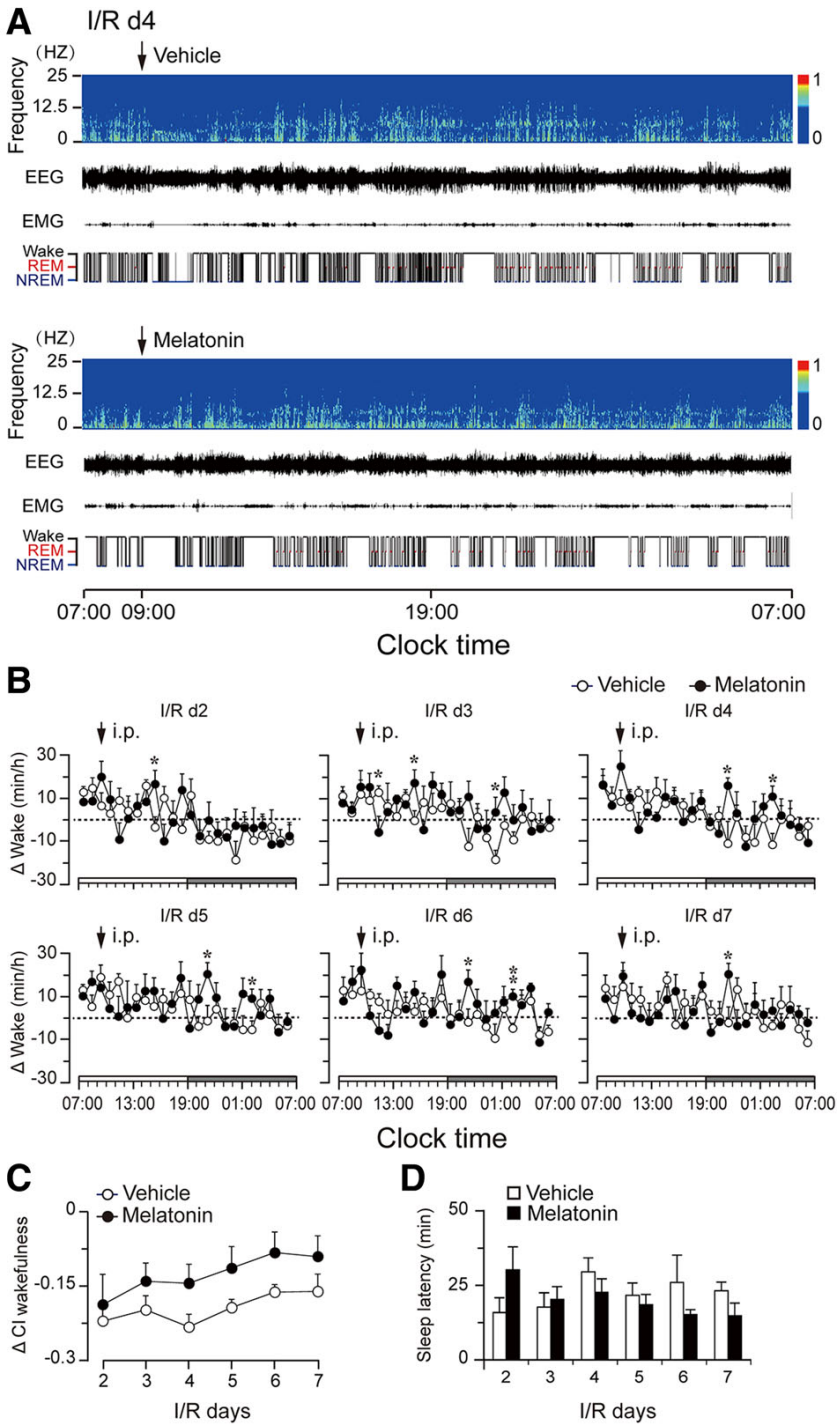
Effects of melatonin on the vigilance states of rats with 30‐min MCAO following different I/R days. (a) Examples of the relative EEG power, EEG/EMG traces, and corresponding hypnograms over 24 h in MCAO rats on I/R d4. (b) Changes in wakefulness (Δ wake = treatment wake – baseline wake) in ischemic rats after vehicle or melatonin treatment during I/R d2–d7. Each circle represents the hourly mean amount of Δ wake. The horizontal open and filled bars on the *x*‐axis indicate 12‐h light and 12‐h dark periods, respectively. (c) The changes in the CI of wakefulness (Δ CI_wakefulness _= treatment CI_wakefulness_ – baseline CI_wakefulness_) in ischemic rats after vehicle or melatonin treatment on I/R d2–d7. (d) NREM sleep latency of ischemic rats after melatonin or vehicle administration at 9:00 am, on I/R d2–d7. Data are presented as the mean ± SE, *n *= 6. *p < 0.05, assessed by two‐tailed unpaired Student's *t*‐test

To investigate the effects of melatonin on the sleep‐wake cycles of rats after ischemic stroke, rats were intraperitoneally administered melatonin or vehicle at 9 a.m. (2 h after light onset) each day for 6 days, from I/R d2 to I/R d7. EEG/EMG recordings were simultaneously obtained. Typical examples of the relative EEG power, EEG/EMG traces, and corresponding hypnograms over 24 h from a vehicle‐treated rat and a melatonin‐treated rat on I/R d4 are shown in Figure 3a. Furthermore, Figure 3b shows the difference in wakefulness (Δ wake) between each treatment and the corresponding baseline level (Figure S2; the baseline EEG of each rat was analyzed, and those with normal sleep‐wake circadian rhythms were included in the analysis). During the light phase, the Δ wake at each time point (per hour) in the vehicle group was above the zero level from I/R d2 to I/R d6. By contrast, during the dark phase, the Δ wake values were mostly negative, especially from I/R d2 to I/R d4. This finding indicates that MCAO induced an increase and a decrease in wakefulness during day and night, respectively, which resulted in a flattened diurnal amplitude of the circadian rhythm (i.e., a decrease of CI in MCAO rats) (Figure S3). We also calculated the changes of CI of wakefulness of each group on every experimental day, as shown in Figure 3c. Starting from the second intraperitoneal administration, the degree of diurnal amplitude reduction in the melatonin group was less severe than that of the vehicle group, suggesting that melatonin may relieve circadian rhythm disturbances in ischemic rats. However, melatonin had no significant effect on nonrapid eye movement (NREM) sleep latency in ischemic rats (Figure 3d).

**FIGURE 4:**
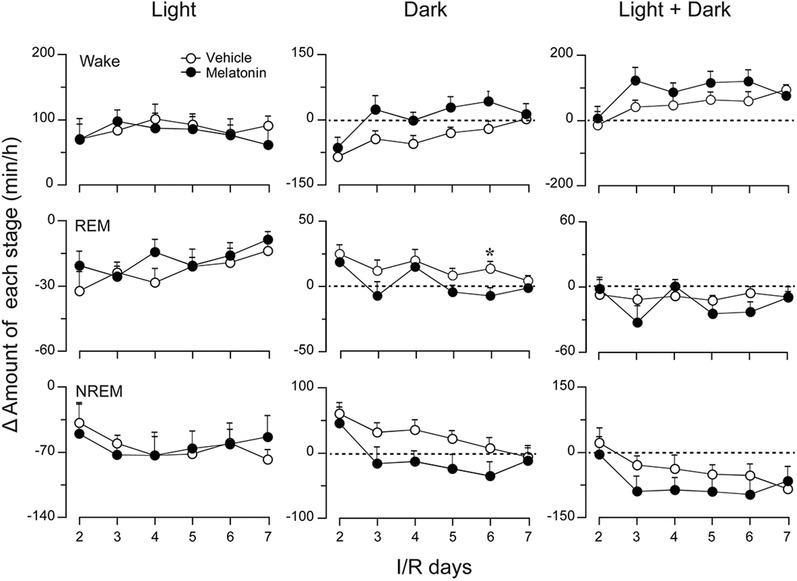
Effects of melatonin on changes in the amounts of each sleep/wake stage in rats with 30‐min MCAO following different numbers of I/R days. Total Δ amounts of wakefulness, REM sleep, and NREM sleep during the light period, dark period, and over the 24‐h period of each I/R day, with daily administration of vehicle or melatonin at 9 a.m. Data are presented as the mean ± SE, *n* = 6. *p < 0.05, assessed by two‐tailed unpaired Student's *t*‐test

Changes in total wakefulness, rapid eye movement (REM) sleep, and NREM sleep amount during the day, night, and a full 24 h cycle are illustrated in Figure 4, further demonstrating the effects of 30 min MCAO on vigilance states and circadian rhythms of MCAO rats (i.e., an increase and a decrease in wakefulness during the day and night, respectively) (Figure 4, left and middle columns). The total amount of Δ wake during the light phase showed no significant difference between vehicle‐ and melatonin‐treated ischemic rats (Figure 4, left column, upper panel). During the dark phase, instead of a decrease in the vehicle group, the total amount of wakefulness of melatonin‐treated ischemic rats was close to the corresponding baseline level since I/R d3, after the second administration of melatonin (Figure 4, middle column, upper panel); this indicates that melatonin quickly and effectively promoted recovery from circadian disturbances in ischemic rats.

### 3.3 Effects of melatonin on sleep characteristics of MCAO rats

**FIGURE 5:**
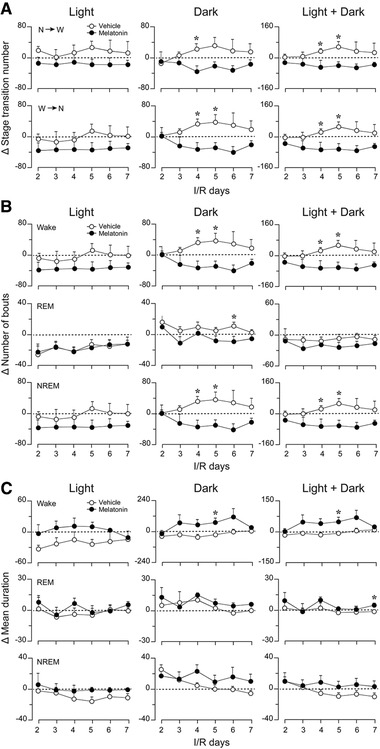
Effects of melatonin on the characteristics of sleep‐wake episodes in rats with 30‐min MCAO following different number of I/R days. (a) Total amount of Δ NREM sleep to wake transitions (upper panel) and Δ wake to NREM sleep transitions (lower panel) during the light period, dark period, and over the 24‐h period of each I/R day, with daily administration of vehicle or melatonin at 9 a.m. (b) Total Δ number of wakefulness, REM sleep, and NREM sleep bouts during the light period, dark period, and over the 24‐h period of each I/R day, with daily administration of vehicle or melatonin at 9 a.m. (c) Δ mean durations of wakefulness, REM sleep, and NREM sleep during the light period, dark period, and over the 24‐h period of each I/R day, with daily administration of vehicle or melatonin at 9 a.m. Data are presented as the mean ± SE, *n* = 6. *p < 0.05, assessed by two‐tailed unpaired Student's *t*‐test

To further investigate the effects of melatonin on sleep‐wake profiles of ischemic rats, we calculated the stage transitions, bout numbers, and mean durations of wakefulness, REM sleep, and NREM sleep on each experimental day, and analyzed the changes in these indicators of sleep (treatment value – corresponding baseline value). As shown in Figure 5a, especially during the dark period, both the changes in transitions from NREM sleep to wakefulness and those of wakefulness to NREM sleep in the melatonin group were less than those of the baseline levels starting at day 2 after drug administration. In contrast, during the same period, these changes in the vehicle ischemic group showed the opposite trend. However, there was no significant change in the number of transitions from NREM sleep to REM sleep or that from REM sleep to wakefulness (data not shown). The changes in the numbers of bouts of wakefulness and NREM sleep were consistent with the changes in stage transitions described above (i.e., melatonin decreased the number of stages [primarily wakefulness and NREM sleep] from I/R d3 to I/R d7 when melatonin was administered daily) (Figure 5b). On the contrary, the changes in the mean duration of wakefulness in the melatonin group were above the baseline level from I/R d3 to I/R d6 (Figure 5c). The reductions in stage transitions and stage bouts, together with increases in stage durations, indicated that melatonin alleviated sleep fragmentation in ischemic rats.

**FIGURE 6:**
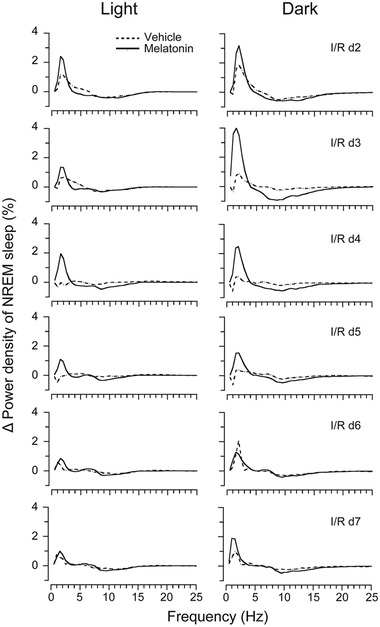
Effects of melatonin on EEG power density during NREM sleep in rats with 30‐min MCAO following different numbers of I/R days. Δ EEG power density of NREM sleep during light and dark periods of each I/R day, with daily administration of vehicle or melatonin at 9 a.m. The changes in power in each frequency bin were obtained by subtracting the corresponding baseline value from the treatment value. Data are presented as the mean ± SE, *n* = 6

Next, we tested whether melatonin affected the EEG power spectra of MCAO rats. We found that after 30 min of MCAO, the NREM sleep power of the vehicle‐treated rats in the delta range (0.5−3.0 Hz) was notably increased on I/R d2 and slightly increased on I/R d3. Starting at I/R d4, the NREM sleep power returned to the baseline level. Daily administration of melatonin at 9 a.m. further increased the NREM sleep delta power on I/R d2 and I/R d3, and this effect lasted until I/R d5, during both the light and dark periods (Figure 6).

## 4 DISCUSSION

To the best of our knowledge, this study is the first to elucidate the effects of melatonin on sleep disorders of transiently ischemic rats. We found that 30‐min MCAO induced disturbances in sleep‐wake circadian rhythms and sleep fragmentation in rats. Moreover, daily melatonin administration during the subacute phase of stroke not only reduced infarct volume and restored brain function but also ameliorated ischemia‐induced sleep disturbances.

Two previous rodent studies have investigated the effects of stroke on sleep. Baumann et al. (2006) induced focal cerebral ischemia in mice and found that striatal stroke led to an increase in NREM sleep and a reduction in REM sleep during both the light and dark phases, and these changes persisted for 12 days after stroke. In contrast, cortical stroke has been shown to induce a smaller increase in NREM sleep, which is present only during the dark phase at day 1 after stroke. More recently, Ahmed et al. (2011) induced a more severe form of stroke (combined cortical/subcortical cerebral ischemia) in rats and reported cerebral‐ischemia‐induced changes in sleep, including a substantial suppression of REM sleep. Our results demonstrated an increase in NREM sleep following MCAO, which was present only during the dark phase in the first 2 days after stroke. In contrast, during the light phase, both NREM sleep and REM sleep were suppressed from I/R d2 to d7. There was no significant difference in the total wake time or sleep time, except for a modest suppression of NREM sleep on I/R d7 after MCAO (Figure S4). The differences among our results and those of previous studies may be due to the different models of stroke that were used and the different degrees of ischemic injury. The infarcts that we induced were less extensive than those reported in Ahmed et al. (90‐min MCAO) and involved both subcortical and cortical infarctions, in contrast to cortical or striatum infarctions in Baumann et al. Our study also revealed a significant increase in NREM sleep delta power during both the light and dark phases on I/R d2−3 (Figure 6), which is consistent with the findings of Ahmed et al. (2011).

In agreement with human data on the impact of stroke on sleep/wake cycles (Hepburn et al., 2018), one of the most remarkable sleep‐wake alterations of MCAO rats in our study was the flattened circadian rhythm, reflected by a reduction in the CI of wakefulness (Figure 3 and S3). However, the mechanisms underlying the observed sleep/wake circadian‐rhythm changes in the MCAO rats in our study and those of human poststroke patients remain unclear. Kinouchi et al. (1994) demonstrated that changes in gene expression can occur in regions remote from the infarction. Hence, we speculate that, in addition to the caudate/putamen and parietal cortex—two structures that are the most consistently affected in this model of stroke—hypothalamic neurons in the suprachiasmatic nucleus (SCN) may also be affected by MCAO injury. However, this hypothesis needs to be tested via future studies on gene transcription or expression of immediate early genes (e.g., c‐fos) in the SCN after ischemic injury.

Melatonin is the most accurate marker of circadian rhythms and is secreted rhythmically under the control of the SCN (Adamczak‐Ratajczak et al., 2017). Although we did not measure the melatonin levels of MCAO rats in the present study, lowered concentrations of melatonin and its metabolite, 6‐sulfatoxymelatinon, in serum and urine have been reported in stroke patients (Adamczak‐Ratajczak et al., 2017; Fiorina et al., 1999; Ritzenthaler et al., 2013). Therefore, the disrupted sleep/wake circadian rhythms that we observed in MCAO rats may have partially resulted from a decrease in melatonin after ischemic injury, suggesting a therapeutic effect of exogenous supplementation of melatonin on sleep disturbances after stroke. In the present study, with daily administration of melatonin (10 mg/kg) for 6 consecutive days, we ascertained that melatonin ameliorated circadian rhythm disturbances (Figure 3 and 4) and alleviated sleep fragmentation in MCAO rats (Figure 5). Moreover, we found that melatonin further increased the power density of NREM sleep on I/R d2–d5 after MCAO, during both the light and dark periods (Figure 6). During the initial stage after MCAO, we also observed increases in NREM sleep and delta power density (Figure 6 and S4). As sleep is associated with plasticity after brain damage (Facchin et al., 2020; Murphy & Corbett, 2009), this phenomenon may be a compensatory response to ischemic brain injury. Although melatonin did not increase the time that MCAO rats spent in NREM sleep, it further enhanced NREM sleep delta power, which corresponds to sleep depth, strongly suggesting a role of exogenous melatonin in improving the sleep quality of MCAO rats.

There have been numerous preclinical studies on melatonin treatment in stroke models that have revealed that melatonin exerts crucial functions including reducing infarct size, edema, inflammatory/apoptotic responses, and mortality, as well as improving neuronal survival and neurogenesis (Andrabi et al., 2015; Balduini et al., 2012; Ramos et al., 2020; Xie et al., 2017; Yang et al., 2015). These beneficial effects are also accompanied by improvements in multiple functional outcomes. Our study yielded similar results, such as a significantly reduced ischemic infarct volume and improved sensorimotor outcomes following melatonin treatment in MCAO rats (Figure 1). Furthermore, we found that Fluoro‐Jade B staining in the contralateral corpus callosum was significantly reduced, and SMI32 immunoreactivity (neuronal cytoskeletal marker) was less intense in the ipsilateral ischemic brain of the melatonin‐treated ischemic group compared to the vehicle‐treated ischemic group (Figure 1b, c and 2), which indicated alleviation in axonal degeneration after melatonin treatment. A cross‐sectional study showed reduced integrity of right lateralized white matter in patients with primary insomnia (Li et al., 2016), suggesting an association between sleep and white matter integrity. Therefore, the reduction in the degeneration of the contralateral corpus callosum in the ischemic group treated with melatonin may have been partly due to a melatonin‐induced reduction in sleep disturbances and improvement in sleep quality.

Although it has been recognized that sleep disturbances increase stroke risk and deteriorate poststroke recovery (Bassetti et al., 2020; Duss et al., 2017; Hermann & Bassetti, 2009; Sterr et al., 2018), sleep problems are still insufficiently considered in the prevention and management of stroke patients (Boulanger et al., 2018; Hepburn et al., 2018; Powers et al., 2019; Sall et al., 2019). Hence, it is necessary to incorporate treatments aimed at sleep disorders (e.g., insomnia) in stroke rehabilitation practices. Melatonin is an alternative treatment to the currently available pharmaceutical therapies for sleep disorders, with significantly fewer side effects (Xie et al., 2017). In our study on MCAO rats, we found that in addition to reducing ischemic damage, melatonin also alleviated MCAO‐induced sleep disturbances, indicating that melatonin may be an appropriate therapeutic drug for clinical stroke rehabilitation. Moreover, melatonin levels decrease with age (Leger et al., 2004; Xie et al., 2017 ), so melatonin may be considered as a daily health‐care drug for the elderly to prevent the incidence of stroke. Therefore, retrospective studies of the elderly population with daily melatonin supplementation are needed to assess the association of melatonin treatment with the incidence of stroke in elderly individuals.

The limitations of the present study include a lack of experiments to reveal the causes of circadian rhythm disturbance in ischemic rats and the correlation between the effects of melatonin on postischemic sleep disturbances and ischemic injury in rats. In addition, the underlying mechanism by which melatonin ameliorates circadian rhythm disruption in ischemic rats needs to be further investigated.

In summary, we found that melatonin supplemented during the sub‐acute stage of stroke mitigated sleep disturbances, and improved the sleep quality and functional outcomes, in rats after transient cerebral ischemia. Given its physiological properties and multiple effects on neuroprotection against ischemia injury, as well as its ability to promptly reverse ischemia‐induced sleep disturbances, melatonin may represent a preferable strategy for improving clinical outcomes in acute stroke victims.

## CONFLICTS OF INTEREST

The authors declare that the research was conducted in the absence of any commercial or financial relationships that could be construed as a potential conflict of interest.

### PEER REVIEW

The peer review history for this article is available at https://publons.com/publon/10.1002/brb3.2366

## Supporting information

Supporting Information

## Data Availability

The data that support the findings of this study are available from the corresponding author upon reasonable request.
