# Supplementary material for: Melatonin supplementation in the subacute phase after ischemia alleviates postischemic sleep disturbances in rats
Source: Brain Behav. 2021 Sep 14;11(10):e2366. doi: 10.1002/brb3.2366 (PMC8553311; doi:10.1002/brb3.2366)
Supplement: Supplementary file 1 — Supporting Information [file BRB3-11-e2366-s001.docx]

**Supplementary Material**

**Melatonin supplementation in the sub-acute phase after ischemia alleviates post-ischemic sleep disturbances in rats**

Shu-Mei Hao^1^, Zhi-Gang Zhong^1.2^, Wei-Min Qu^2^, Zhi-Li Huang^2^, Feng-Yan Sun^1^, Mei-Hong Qiu*^1.2^

^1^Department of Neurobiology, Institute for Basic Research on Aging and Medicine, School of Basic Medical Science, Fudan University, Shanghai, China.

^2^Department of Pharmacology, School of Basic Medical Science, State Key Laboratory of Medical Neurobiology and MOE Frontiers Center for Brain Science, Fudan University, Shanghai, China.

**Expanded materials & methods:**

**EEG/EMG recordings and analysis**

Six days after implantation of the electrodes, each rat was transferred to the recording room to habituate to the recording cable and conditions for 1 day. Then, the EEG and EMG signals were continuously recorded for 48 h (serving as baseline sleep data). Each rat was then subjected to transient MCAO for 30 min, and EEG/EMG recordings were obtained from 2 to 7 days after ischemic stroke. The recordings were started at 7:00 a.m. (onset of light period). EEG/EMG signals were amplified and filtered (EEG, 0.5–30 Hz; EMG, 20–200 Hz), digitized at a sampling rate of 128 Hz, and recorded using VitalRecorder (Kissei Comtec, Nagano, Japan). When completed, EEG/EMG data were automatically scored offline in 10-s epochs as wakefulness, non-rapid eye movement (NREM) sleep, or rapid eye movement (REM) sleep in SleepSign (Kissei Comtec, Nagano, Japan) using standard criteria ^40^. After automatic scoring, the sleep-wake stages were examined and manually corrected. The durations spent in wakefulness, NREM sleep, and REM sleep were determined from the scored EEG/EMG data. EEG power spectra for each epoch were analyzed offline using fast Fourier transformation (256 point, Hanning window, 0–24.5 Hz with 0.5-Hz resolution using SleepSign).

**Transient focal ischemia**

Transient focal ischemia was produced by 30 min of MCAO. The rats were anesthetized by intraperitoneal injection of 10% chloral hydrate at a dose of 360 mg/kg. Body temperature was monitored via a rectal probe and was maintained at 37°C ± 0.5°C throughout the surgeries using a heating pad. Arterial blood samples were collected to measure pCO_2_, pO_2_, and pH via an i-STAT blood-gas analyzer (Abbott Laboratories, Chicago, USA). Rats with normal physiological indicators were subjected to MCAO surgery. MCAO was performed mainly according to the method described by Longa et al. ^41^. In brief, the left common carotid artery, external carotid artery (ECA), and internal carotid artery (ICA) were isolated. A 4-0 nylon monofilament was introduced into the ECA lumen and was gently advanced into the ICA until slight resistance was felt. The filament was left in place for 30 min before being withdrawn to allow reperfusion of the ischemic brain. After recovering from anesthesia, rats were returned to the recording chamber for EEG/EMG acquisition, during which time, they were provided food and water *ad libitum*. The mortality rate following 30 min MCAO in the present study was 7.9% (3 out of 38 rats).

**Inclusion and exclusion criteria**

After the MCAO surgery and recovery from anesthesia, all rats except those with neurological scores^41^ of 0 (2 out of 35), underwent subsequent treatments. The baseline EEG of each rat was analyzed first, and those with normal sleep-wake circadian rhythms were included in the analysis.

**Fluoro-Jade B staining**

Fluoro-Jade B staining was used to reveal the infarct area of each ischemic brain. In brief, brain sections from Bregma +2.0 mm to -2.0 mm, as per the atlas of Paxinos and Watson ^42^, were immersed in the following: 80% ethanol containing 1% sodium hydroxide for 5 min; 70% ethanol for 2 min; 0.06% potassium permanganate for 10 min; and Fluoro-Jade B (4 mg/L in 0.1% acetic acid, Millipore, Billerica, MA, USA) for 20 min. The fluorescent signals were detected at an excitation wavelength of 488 nm via an Olympus VS120 slide-scanner microscope. The infarct area of each section and the length of the contralateral corpus callosum were measured and calculated using ImageJ software (National Institute of Health, Bethesda, MD, USA). The infarction volumes are expressed as a percentage of the volume of the contralateral hemisphere, as previously described ^43^.

**Immunohistochemical staining**

Following incubation with a blocking solution for 1 h at 37°C, the sections were incubated with 0.3% H_2_O_2_ for 15 min to quench endogenous peroxidase activity, after which they were incubated with a mouse primary antibody against SMI32 (1:500; ab27375, Abcam, Cambridge, UK) overnight at 4°C. On the second day, the sections were washed and incubated with biotinylated anti-mouse IgG (1:200; Vector Laboratories Inc., Burlingame, CA, USA) at 37°C for 1 h, followed by incubation with an avidin-biotin-peroxidase complex (Vector Laboratories Inc., Burlingame, CA, USA) for 1 h at room temperature. The peroxidase reaction was visualized with 0.05% 3, 3-diaminobenzidine tetrahydrochloride (Sigma-Aldrich, St. Louis, MO, USA) solution containing 0.03% H_2_O_2_. The brain slices were then mounted, dehydrated, and cover-slipped. As controls, adjacent sections received an identical treatment but without incubation in the primary antibody to confirm that no non-specific stanning had occurred. Immunostained images were captured with an Olympus VS120 slide-scanner microscope. To evaluate the axonal injury after MCAO, the SMI32-imunopositive axon fascicles within the area of 0.91 mm × 0.53 mm in the infarct striatum were quantitatively analyzed using ImageJ software on three adjacent coronary brain slices per rat. The average quantification of each rat was used to represent the data.

**Wire hang test**

On day 8 after the MCAO operation, the wire hang test was used to assess the motor function of each rat, particularly muscle strength, endurance, and grip strength. Each rat was placed on a wire cage lid (30-cm width × 45-cm length) and was allowed to grasp the wire cage with all four limbs. Then, the cage was turned upside down, during which each rat hung on the top of the cage. The time until each rat fell from the top of the cage was recorded as the latency to falling. The procedure was repeated five times, with intervals of 15 min between each session. The average falling latency of the five trials was taken as the test data ^44^.

**Cylinder test**

The cylinder test was used to evaluate the locomotor asymmetry of forelimb usage of each rat. On day 8 after the MCAO operation, each rat was placed individually in a transparent cylinder (30 cm in inner diameter and 35 cm in height), and the number of times that the rat touched the wall with either forepaw was counted. A total of 20 touches were recorded for each rat. The cylinder score for the impaired forelimb was calculated as follows: (Impaired paw + ½ both paws)/ (total touches) *100% ^45^.

**Rotarod test**

The rotarod test was used to evaluate the motor coordination and balance of each rat. The rats were trained on day 8 and 9 after MCAO and were tested on day 10 after MCAO. Rats were placed on a rotarod (Med Associates, Georgia, VT, USA) accelerating from 4 to 40 rpm over a 5-min period. Each trial started at the onset of acceleration and ended when the rat fell off the rotarod. The procedure was repeated for three trials, each separated by a 15-min inter-trial interval. The latency to fall from the rotarod of each trial was recorded, and the test data are presented as the mean latency of the three trials.

**Supplementary figures and figure legends:**

**Figure S1:**


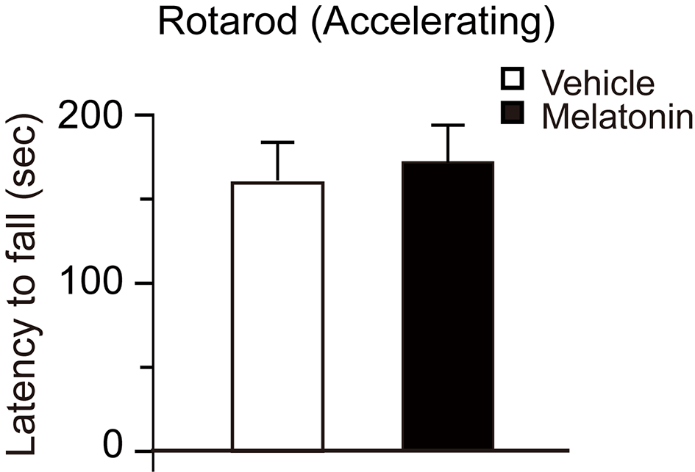


**Figure S1.** **Sensorimotor coordination of rats with vehicle or melatonin treatments on day 10 after MCAO.**

There was no significant difference in the behavioral performance on the rotarod test between the two groups at I/R d10. Data are presented as the mean ± SE, n = 8.

**Figure S2:**


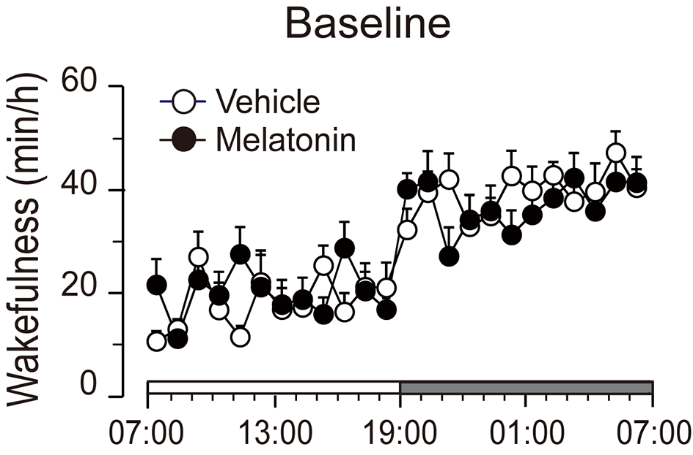


**Figure S2.** **Baseline awakening time of rats before the MCAO procedure.**

Hourly time courses of wakefulness across 24 h of the rats used in Figure 3b. These rats exhibited normal circadian patterns of sleep-wake behavior, being more active during the dark period (active phase) and exhibiting more sleep during the light period (inactive phase). Data are presented as the mean ± SE, n = 6.

**Figure S3:**


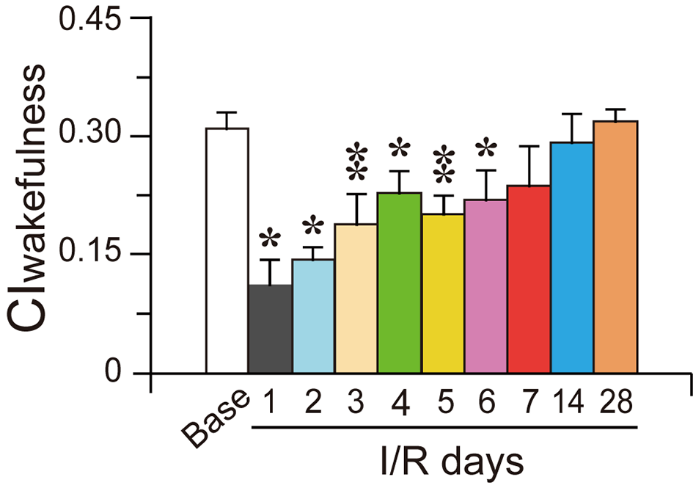


**Figure S3.** **Effect of ischemia on circadian rhythms in rats.**

Circadian index of wakefulness of rats on I/R d1–7, I/R d14, and I/R d28 after 30-min MCAO. The data show that 30-min MCAO significantly reduced the CI of wakefulness of rats on the first reperfusion day after ischemia, and the effect lasted for 6 days, indicating that ischemic stroke rapidly resulted in circadian rhythm disturbances. Data are presented as the mean ± SE, n = 6. **p* < 0.05, ***p* < 0.01, assessed by two-tailed paired Student’s *t*- test.


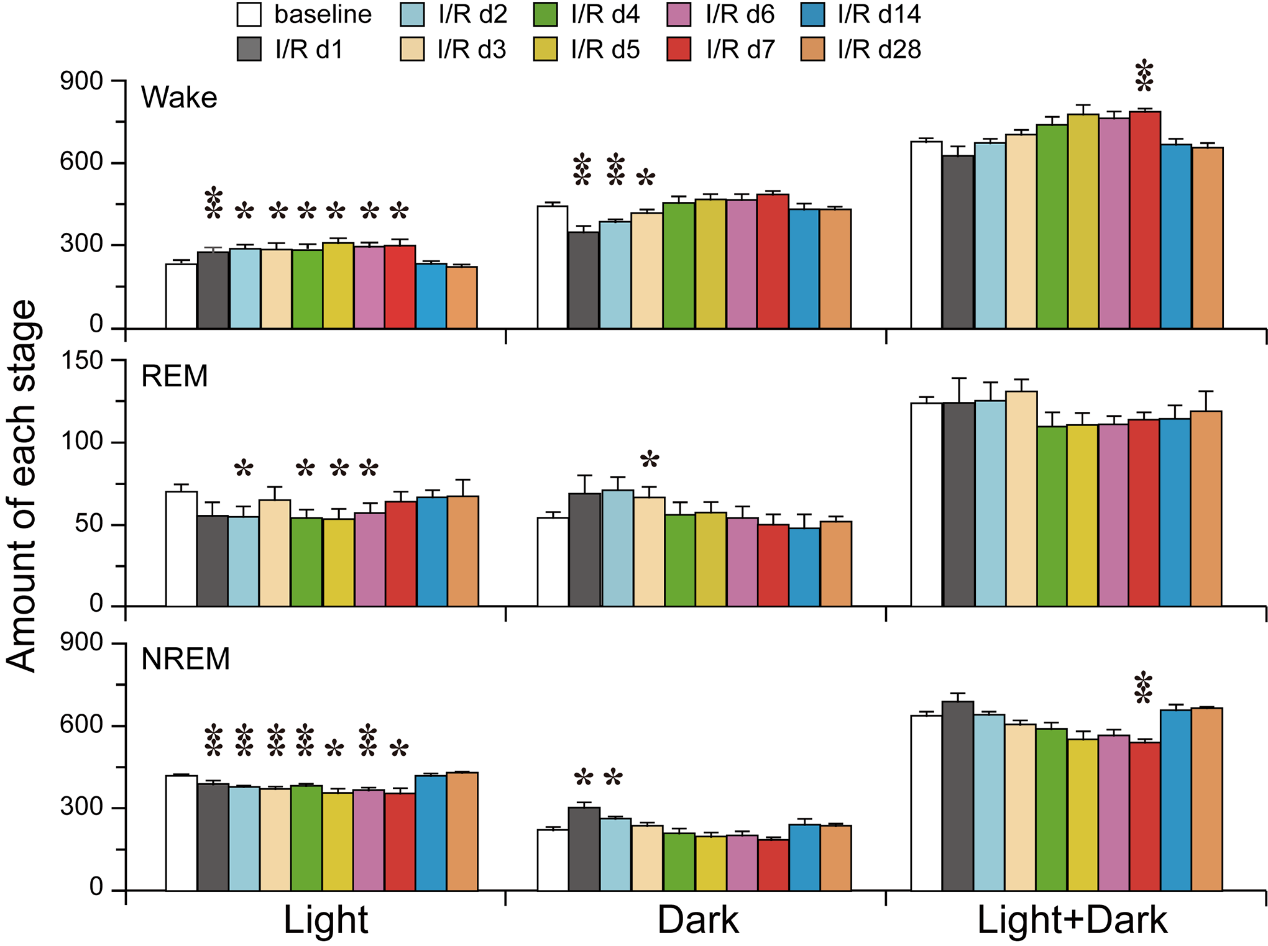
 **Figure S4:**

**Figure S4.** **Changes in wakefulness, REM sleep, and NREM sleep amount in rats with 30-min MCAO following different numbers of I/R days.**

Total amounts of wakefulness, REM sleep, and NREM sleep during the light period, dark period, and over each 24-h period of rats before and after ischemia. Data are presented as the mean ± SE, n = 6. **p* < 0.05, ***p* < 0.01, assessed by two-tailed paired Student’s *t*- test.
